# The Chattahoochee Valley Medical Association

**Published:** 1916-05

**Authors:** 


### EDITORIAL

The Atlanta Journal-Record of Medicine has its Business Office at 23 West Alabama Street where all business communications should be sent.

Remittances are payable to The Journal-Record of Medicine.

Concerning Original Communications and Editorial matters, address Richard R. Daly, M. D., 708 Empire Life Building, Atlanta, Ga.

Reprints of Original Articles will be furnished at cost price. Requests for them had best be made on the Manuscript.

Upon request, we will present, postpaid, to each contributor of an original article, twenty copies of The Journal-Record of Medicine containing such article.

### THE CHATTAHOOCHEE VALLEY MEDICAL ASSOCIATION.

We are believers in medical societies when they are well conducted. That last phrase means simply that the plans are such that each man feels he gets something for his membership and that he has an equal chance with any of his fellows to participate in the proceedings of the organization.

The Chattahoochee Valley Association is a get together organization of men who have to know each other because of the contiguity of their work. It has been so guided by the beneficent influence of its Secretary, Dr. W. J. Love, of Opelika, Ala., that its members enjoy its meetings and all feel the spirit that makes a society live and be of value in the world. In it the city man meets his country brother and learns some of the conditions that are beyond his observation yet which have their bearing upon the patients that may come to him for special treatment. There the rural man can acquire information at first hand as to the new conditions the city clinics have but recently demonstrated, and at the same time each man can get a personal impression of the other that will be of use later on when consultations are needed. There is a refreshing breadth of view acquired every time such meetings occur when by the recounting of experiences in the varying fields of work each man learns to know his neighbor better and discovers details of methods that are invaluable to him.

The next meeting of the Association will occur in July at Alexander City, Alabama, under the presidency of Dr. Hugh M. Lokey, of Atlanta. A very interesting program is being arranged and the titles show the great importance the men assign to this meeting. As soon as the program is completed it will be published here.

Meantime Atlanta men are asked to communicate with Dr. Lokey so that private sleepers can be secured for the trip. A large attendance is sought and we are assured not only by the papers thus far submitted but also by the plans underway by the Alexandrians that the meeting will be a success. It is suggested that special car arrangements be made in every town wherefrom many men are going to the meeting. Members along the route from Atlanta to Alexandria might well communicate with Dr. Lokey, Candler Building, Atlanta, as to transportation plans.

---

Editor Journal-Record of Medicine,

Dear Doctor:

The Chattahoochee Valley Medical and Surgical Associatio will hold its meeting this year at Alexander City, Ala., July 11th and 12th, next.

The coming meeting of this most progressive association will have many features of more than usual interest. Ten years ago it was organized in this beautiful little city with only seventeen members, and this is the first time it has returned to hold its session in this, its parent city, and it will be with pride that its charter members will look upon its growth and influence, for now its membership is near the three hundred mark. Its influence for good in the medical and surgical profession is both seen and felt.

It is the one medical association that is free of politics—in it every member stands upon his own merits, there are no favorites, nor scheming—but an organization of medical men who have the upbuilding of the medical profession at heart, and whose aim is to raise it far above the plain of petty professional strife by cultivating each other, and raising, and inculcating higher ideas of professional dignity. Indeed, one has only to attend its meetings to be wonderfully impressed with the personel of its members, the hale fellow well met cordiality, the hearty warm hand clasp, and the feeling of professional brotherhood that prevails at its meetings; and, if it is the first time that you have attended one of its meetings, you will be surprised at the ultra scientific spirit that characterizes the papers read, and the great thoroughness of the discussions.

It has been said, and often repeated, that nowhere are medical topics so completely thrashed out, and so thoroughly discussed, as on the floor of the Chattahoochee Valley Medical and Surgical Association. They are all a busy, jolly set, no drones need apply, for they would not survive. Its membership is truly an exemplification of the "survival of the fittest."

The program of the coming meeting will be large and interesting, and the people of Alexander City and its surrounding towns and villages, not only doctors, but the entire citizenship will bestir themselves to give the association a warm homecoming welcome, one that will be long remembered as one of the very best medical meetings ever held in this part of the country.

Go and take all your friends, the boys will welcome you, and you will be glad you went.

W. J. Love

Opelika, Ala.

